# Effects of fire intensity on soil microbial diversity and nitrogen cycling functional genes in forests (Northeast China)

**DOI:** 10.3389/fmicb.2025.1615520

**Published:** 2025-07-25

**Authors:** Wenjie Jia, Yang Shu, Pengwu Zhao, Mei Zhou, Yongjie Yue

**Affiliations:** ^1^College of Forestry, Inner Mongolia Agricultural University, Hohhot, China; ^2^National Orientation Observation and Research Station of Saihanwula Forest Ecosystem in Inner Mongolia, Chifeng, China

**Keywords:** fire intensity, microbial diversity, nitrogen cycle, gene abundance, *Larix gmelinii* forests

## Abstract

**Introduction:**

Forest fire disturbance is one of the most critical factors affecting forest ecosystems in Northeast China. It disrupts ecosystem balance, alters soil physical and chemical properties, and significantly impacts soil microbial communities and nitrogen cycling. Understanding these changes is essential for post-fire vegetation restoration and nitrogen pool reconstruction.

**Methods:**

This study focused on a burned Larix gmelinii forest in the Daxing’an Mountains. We investigated soil environmental factors, microbial community structure, nitrogen cycle genes, and their interrelationships under different fire intensity conditions.

**Results:**

(1) Light fire increased soil pH, total nitrogen (TN), soil organic carbon (SOC), nitrate nitrogen (NO_3_^–^-N), and available phosphorus (AP), but reduced soil moisture content (SMC), microbial biomass carbon (MBC), microbial biomass nitrogen (MBN), and ammonium nitrogen (NH_4_^+^-N). Severe fire raised bulk density (BD), available potassium (AK), AP, and NO_3_^–^-N, while decreasing SMC, MBC, MBN, NH_4_^+^-N, and TN. (2) Bacterial diversity (Shannon index) increased after light fire but decreased after severe fire; richness indices (Sobs and Chao1) declined under both fire conditions. Fungal diversity and richness declined with both light and severe fires. Dominant soil bacterial phylum was Proteobacteria (with Bradyrhizobium as dominant genus), while dominant fungal phylum was Basidiomycota (with Russula as dominant genus). (3) Abundance of nitrogen fixation gene *nifH* declined with increasing fire intensity. Abundance of nitrification genes *amoA-AOA* and *amoA-AOB* significantly increased. Denitrification genes (*nirK, nirS, nosZ*) increased after light fire but decreased after severe fire. (4) Soil nitrogen (MBN, TN, NH_4_^+^-N, NO_3_^–^-N) had a direct positive effect on nitrogen cycle genes, while fire intensity, available nutrients (AP, AK), and bacterial communities had direct negative effects.

**Discussion:**

The findings reveal the complex response of soil properties, microbial communities and nitrogen cycle genes to different fire intensities. These findings provide a scientific basis for effective post-fire ecosystem management and soil fertility restoration in the boreal forest.

## 1 Introduction

Fire is a common phenomenon in forest ecosystems, with over 200,000 forest fires occurring globally each year, burning tens of millions of hectares or more than 1% of the world’s total forest area (Rogers et al., 2020; Wang, 2023). With global warming, fire-affected areas are projected to increase by 29% (Senande-Rivera et al., 2022) along with increasing fire frequency and intensity (Sheikh et al., 2022). Soil microorganisms, as crucial components of forest ecosystems, play a key role in regulating the material and energy cycles, particularly carbon and nitrogen cycles (Srivastava et al., 2023; Basu et al., 2021). The richness and composition of soil microbial communities serve as key indicators of ecosystem function and soil quality (Li et al., 2008). Forest fires can directly cause microbial mortality and alter microbial biomass, activity, community structure, and function (Holden et al., 2013). Indirectly, fires modify soil properties by burning organic matter, leading to nutrient volatilization and changes in soil texture, pH, water content, and oxygen levels (Martin et al., 2022). Studies have suggested that soil microorganisms can exhibit a more pronounced response to forest fires than soil physicochemical properties (Pressler et al., 2019), with the microbial biomass and abundance decreasing by up to 96% and microbial diversity, richness, and evenness declining by 99%, often persisting for over a decade (Feng et al., 2018). Therefore, fires of different intensities will significantly affect the composition of soil microbial communities, which in turn will affect soil nutrient cycling.

Nitrogen (N) is one of the most important nutrient elements in the ecosystem and has an important impact on plant growth and maintaining the stability of the ecosystem (Caon et al., 2014). The soil nitrogen pool is primarily stored as organic nitrogen, much of which requires microbial processing for plant absorption and utilization (Chen et al., 2014). As a key component of soil and biosphere energy flow, the nitrogen cycle plays a fundamental role in sustaining life and supporting agricultural ecosystems (Doblas-Miranda et al., 2015). It consists of nitrogen fixation, nitrification, and denitrification, involving key nitrogen cycle genes, such as *nifH*, *amoA-AOA*, *amoA-AOB*, *narG*, and *nosZ*. Forest fires are powerful ecological disturbances that strongly alter ecosystem processes and functions (Prendergast-Miller et al., 2017; Lin et al., 2021). Compared with other ecosystems, fires in northern forests cause significant imbalances in soil carbon and nitrogen cycles (Yang S. et al., 2020), with nitrogen being the most affected soil nutrient (Li et al., 2021). Studies have indicated that forest and soil carbon-nitrogen cycles are highly sensitive to fire disturbances, with impacts largely dependent on fire severity, recurrence, and post-fire recovery time (Doblas-Miranda et al., 2015; Pérez-Izquierdo et al., 2021; Pellegrini et al., 2018). Studies by Vourlitis (2017), Kolka et al. (2017), and Pellegrini et al. (2018) have shown that severe fires could significantly alter soil microbial communities and disrupt nitrogen biogeochemical cycles. Fire releases large amounts of nitrogen from aboveground biomass while temporarily increasing available nitrogen, which promotes post-fire vegetation regeneration. However, this increase has typically lasted for only 1–2 years.

The Daxing’an Mountain region serves as a crucial ecological security barrier for northern China and is home to the only bright coniferous forest ecosystem in the cold temperate zone of China (Yang J. et al., 2021). *Larix gmelinii* is the dominant and constructive species in this region, playing a vital role in maintaining forest stability (Li et al., 2022). Domestic and foreign research scholars have carried out relatively single research on the three aspects of soil physical and chemical properties, microbial diversity and nitrogen cycle genes after fire, and the interaction between the three is rarely reported. To protect the forest ecosystems of northern China and assess the short- and long-term effects of different fire intensities on the soil microbial nitrogen cycle, this study conducted field investigation and quantitative PCR analysis in fire-affected areas. This study examined variations in soil physicochemical properties, microbial community diversity, and abundance of nitrogen cycle-related genes under different fire intensities. Additionally, the interactions among soil environmental factors, microbial communities, and nitrogen cycle genes were analyzed to provide a theoretical basis for soil nitrogen pool reconstruction, rapid vegetation regeneration, and post-fire ecosystem restoration.

## 2 Materials and methods

### 2.1 Overview of the study area

**FIGURE 1:**
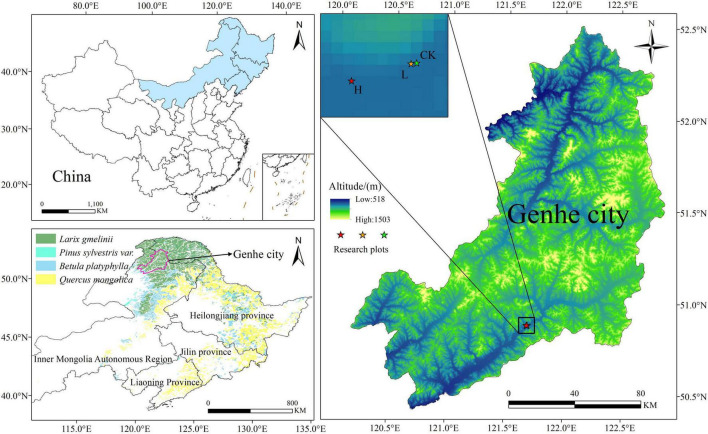
Overview map of the study area [vegetation type data from the literature (Zhang, 2007)].

The study area is located in Genhe City, Inner Mongolia Autonomous Region (120°41′30″–122°42′30″E, 50°25′30″–51°17′00″E) (Figure 1), and the terrain is high in the northeast and low in the southwest, averaging an altitude of approximately 1,000 m. It lies in the cold temperate zone and experiences a typical cold temperate continental climate, with annual precipitation ranging from 450 to 550 mm, an average annual temperature of −5.3°C, and a freezing period lasting up to 9 months. The predominant soil type is brown coniferous forest soil. The region has abundant forest resources, covering a total area of 174.5 ha with a high forest coverage rate of 91.7%. The primary vegetation consists of *Larix gmelinii*, *Betula platyphylla*, *Pinus sylvestris*, *Rosa davurica*, *Ledum palustre*, and *Rhododendron dauricum* (Zhang et al., 2019).

### 2.2 Sample plot setting and sample collection

In September 2022, sample plots were established, and soil samples were collected from the burned area of the Shangyanggeqi Forest Farm, which experienced a lightning-induced fire in 2019, affecting 120.15 ha. Based on the proportion of damaged standing trees, the fire intensity (Lu et al., 2025; Liu et al., 2019) was categorized as unburned (CK), light fire (L), and severe fire (H). To ensure that the fire intensity was the only variable, control plots were established in adjacent areas with identical site conditions. The criteria for plot classification are listed in Table 1. Each 30 m × 30 m plot was replicated three times within each fire intensity category, resulting in nine plots (Table 2). In order to reduce the experimental error, three soil profiles per plot were dug following an “S-type” pattern. Because of the shallow soil layer in this region, the samples were collected from layers A (0–10 cm) and B (10–20 cm), with at least 500 g of soil per layer, following the principle of “first down and then up” while excluding litter and humus (Carter and Gregorich, 2007). After thorough mixing and removal of debris and rhizomes, the soil samples were placed in sterile self-sealing bags, labeled, and stored under refrigeration for transport to the laboratory. Each sample was divided into two portions: one was air-dried and sieved (2 mm) for soil physicochemical analysis, and the other was stored at −80°C for microbial community assessment and nitrogen cycle functional gene analysis.

**TABLE 1:** Characterization of fire intensity classification.

Feature	Fire intensity		
	Unburned	Light fire	Severe fire
Fire disturbance type	–	Surface fire	Surface fire and crown fire
Injured tree	–	≤ 30%	≥ 70%
Blackening height	–	≤ 2 m	≥ 5 m
Severity	–	Understory shrubs were partially burned, and litter was partially burned.	Understory shrubs and litter were all burned down.
Fire interference level	–	The organic matter layer is completely preserved, and the carbonization depth is only a few millimeters.	Ash deposits and charred organic matter up to a few centimeters thick.

**TABLE 2:** Overview of detailed information on fire trails.

Plot type	Altitude	Longitude and latitude	Slope	Slope aspect	Tree height	Diameter at breast height	Vegetation type
Control sample plot	1146.4 m	121°41′45″E — 50°52′54″N	4°	Southwest	21.5 ± 2.1 m	33.1 ± 4.7 m	*Rhododendron dauricum*
Light fire	1130.7 m	121°41′42″E — 50°52′53″N	6°	Southwest	17.5 ± 1.7 m	23.1 ± 6.3 m	*Rhododendron dauricum*
Severe fire	1144.9 m	121°41′19″E — 50°52′49″N	4°	Southwest	15.1 ± 6.9 m	15.9 ± 7.9 m	*Rhododendron dauricum*

### 2.3 Determination of soil physicochemical properties

Soil bulk density (BD) was measured using the ring knife method, while soil moisture content (SMC) was determined using the drying-weighing method. The soil pH was analyzed using the glass electrode method with the water-to-soil ratio of 2.5:1. Nitrate nitrogen (NO_3_^–^-N) and ammonium nitrogen (NH_4_^+^-N) contents were quantified using a flow analyzer, and total nitrogen (TN) was measured using the Kjeldahl method (Lu, 2000). Soil organic carbon (SOC) content was determined using the potassium dichromate oxidation method, whereas microbial biomass carbon (MBC) and microbial biomass nitrogen (MBN) were assessed using the chloroform fumigation method (Brookes et al., 1985). Available phosphorus (AP) was extracted with sodium bicarbonate and analyzed using molybdenum-antimony colorimetry, and available potassium (AK) was determined using ammonium acetate extraction followed by flame photometry (Bao, 2000).

### 2.4 Determination of soil microbial community

According to the research area and the target, the sample collection scheme is designed to ensure the representativeness and uniformity of the sample. According to the manufacturer’s instructions, total DNA was extracted from 0.4 g of soil samples using the E.Z.N.A. ® Soil DNA Kit soil kit (Omega Bio-tek, Norcross, GA, United States), the purity and integrity of DNA were detected by NanoDrop2000 spectrophotometer and 1% agarose gel electrophoresis. PCR amplification was performed on the V3–V4 region of 16S rRNA and ITS genes. Primers 338F, 806R and ITS1F, ITS2R were used to optimize the annealing temperature to improve the amplification efficiency. The PCR products were identified by 2% agarose gel electrophoresis, purified by AxyPrep DNA Gel Extraction Kit (Axygen Biosciences, Union City, CA, United States), and quantified by Quantus TM Fluorometer (Promega, United States) to ensure the homogeneity of the sequencing samples.

When constructing the sequencing library, the NEXTFLEX Rapid DNA-Seq Kit was used for adaptor linking, magnetic bead screening, PCR amplification, and product recovery. The sequencing was completed on the Illumina Miseq PE300 platform, and the sequence was read using bridge amplification technology and fluorescently labeled dNTP. The original data was subjected to quality control by fastp (Chen et al., 2018) (version 0.19.6^1^) software, and the bases with adapter and tail mass values below 20 were removed. FLASH (Magoč and Steven, 2011) (version 1.2.11^2^) was used to identify and remove the primer sequence according to the parameters of allowable maximum mismatch rate of 20% and minimum coverage of 80%. The optimized data were subjected to OTU (operational taxonomic units) clustering and taxonomic analysis of non-repetitive sequences with a similarity of more than 97% by UPARSE (Edgar, 2013; Stackebrandt and Goebel, 1994) (version 7.1^3^), and species annotation was based on the Silva 16S rRNA and ITS gene database. Finally, the OTUs taxonomic annotation results of each sample were obtained.

### 2.5 Determination of functional genes in the soil microbial nitrogen cycle

A 0.4 g soil sample was used for DNA extraction with the NovaSeq Reagent Kit/HiSeq DNA Extraction Kit (Illumina, United States), following the manufacturer’s instructions. DNA purity was assessed using a NanoDrop 2000 spectrophotometer (Thermo Fisher Scientific, United States), and DNA concentration was measured using a TBS-380 microfluorometer (Turner BioSystems, United States). Using the extracted total DNA as a template, specific primers were used to amplify six nitrogen cycle genes involved in soil microbial processes (Table 3). The PCR amplification protocol consisted of an initial denaturation step at 94°C for 5 min, followed by 36 cycles of 95°C for 5 s and 60°C for 60 s. The amplified products were verified using 2% agarose gel electrophoresis and purified. A sequencing library was prepared using the NEXTFLEX Rapid DNA-Seq Kit and metagenomic sequencing was performed on the Illumina NovaSeq platform (Liu and Liu, 2018).

**TABLE 3:** Primer information of nitrogen cycle genes.

Target genes	Primer sequence	Function (Tang et al., 2018)
*nifH*	MMF2: TNATCACCKCNATCACTTCC	N_2_→NH_3_
	MMR1: CGCCGGACKWGACGATGTAG	
*nirS*	Cd3aF: GTSAACGTSAAGGARACSGG	NO_2_^–^→NO
	R3cd:GASTTCGGRTGSGTCTTGA	
*nirK*	nirk876: ATYGGCGGVCAYGGCGA	NO_2_^–^→NO
	nirk1040: GCCTCGATCAGRTTRTGGT	
*nosZ*	nosz2F:CGCRACGGCAASAAGGTSMSSGT	N_2_O→N_2_
	nosz2R: CAKRTGCAKSGCRTGGCAGAA	
*amoA-AOA*	Arch-AmoAF:STAATGGTCTGGCTTAGACG	NH_3_→NH_2_OH
	Arch-AmoAR:GCGGCCATCCATCTGTATGT	
*amoA-AOB*	amoa1F: GGGGTTTCTACTGGTGGT	NH_3_→NH_2_OH
	amoa2R: CCCCTCKGSAAAGCCTTCTTC	

### 2.6 Statistical analysis

Data analysis was performed using Excel 2022 and SPSS 27.0. One-way ANOVA was used to assess the significant differences in the soil physicochemical properties and soil microbial nitrogen cycle gene abundance across different fire intensities, with the LSD multiple comparison (Least Significant Difference) analysis applied for significance testing (*P* < 0.05). Mapping was conducted using Origin 2024, and correlations between soil properties and microbial nitrogen cycle gene abundance were analyzed. Interactive analysis was performed on the Shanghai Majorbio cloud platform^4^ to compute the microbial community composition, α diversity, β diversity (PCoA), soil properties, and microbial correlations based on OTUs using correlation analysis and plotting. Adobe Acrobat 2024 was used for picture text editing. Additionally, the “plspm” package (Ciavolino et al., 2023) in R (version 4.0) was used to construct the path model, and SigmaPlot 12.5 was employed for visualization.

## 3 Results

### 3.1 Soil physicochemical properties

Significant differences in the soil physicochemical properties were observed across plots with varying fire intensities (Table 4). Compared with the unburned plots at 0–20 cm depth, soil pH, BD, NO_3_^–^-N, and AP increased by 4.41% (*P* < 0.05), 5.22% (*P* < 0.05), 1.38%, and 16.22% (*P* < 0.05) in the light fire plots and by 10.19% (*P* < 0.05), 40.69% (*P* < 0.05), 25.82% (*P* < 0.05), and 41.63% (*P* < 0.05) in the severe fire plots, with these values increasing with fire intensity. The contents of SMC, MBC, MBN and NH_4_^+^-N in soil decreased by 3.04%, 7.11%, 12.94% (*P* < 0.05) and 11.08% respectively after light fire, and increased by 3.99% (*P* < 0.05), 17.32% (*P* < 0.05), 9.12% and 19.44% respectively after severe fire. Except for MBN, the contents of SMC, MBN and NH_4_^+^-N decreased with the increase of fire intensity. The contents of TN and SOC increased by 25.64% (*P* < 0.05) and 37.65% (*P* < 0.05) in the light burned plots, respectively, and decreased by 17.68% (*P* < 0.05) and 6.48% in the heavy burned plots, respectively. In contrast, the AK content exhibited the opposite trend, decreasing by 2.90% in the light fire plots but increasing by 19.33% (*P* < 0.05) in the severe fire plots. The significance analysis indicated that more indices demonstrated significant differences in both the 0–10 cm and 10–20 cm soil layers in the severe fire plots than in the light fire plots, both of which exhibited more variation than in the unburned plots. These findings suggest that severe fires induce pronounced changes in the physicochemical properties of soil.

**TABLE 4:** Effects of different fire intensities on the physicochemical properties of soil.

Physicochemical properties	Soil depth (cm)	Fire intensity		
		Unburned	Light fire	Severe fire
pH	0–10	5.04 ± 0.14Ac	5.30 ± 0.26Ab	5.60 ± 0.13Aa
	10–20	5.05 ± 0.19Aab	5.23 ± 0.18Aa	5.01 ± 0.07Bb
BD (g/cm^3^)	0–10	0.83 ± 0.08Ab	0.86 ± 0.03Ab	1.04 ± 0.13Aa
	10–20	0.86 ± 0.03Ab	0.85 ± 0.04Ab	0.92 ± 0.03Ba
SMC (%)	0–10	41.30 ± 1.33Aa	38.81 ± 1.18Bb	38.72 ± 1.26Bb
	10–20	41.42 ± 1.55Aa	41.28 ± 1.16Aa	40.70 ± 1.59Aa
TN (g/kg)	0–10	3.86 ± 0.28Ab	4.74 ± 0.33Aa	2.99 ± 0.14Ac
	10–20	3.31 ± 0.39Bb	4.26 ± 0.29Ba	2.91 ± 0.13Ac
AP (mg/kg)	0–10	21.58 ± 2.94Ab	23.10 ± 3.61Ab	27.73 ± 2.29Aa
	10–20	12.49 ± 1.84Bb	19.77 ± 1.74Ba	20.54 ± 1.18Ba
AK (mg/kg)	0–10	237.92 ± 11.74Ab	251.82 ± 35.30Ab	305.43 ± 20.21Aa
	10–20	233.39 ± 24.69Aab	205.81 ± 27.43Bb	256.99 ± 29.24Ba
SOC (g/kg)	0–10	91.04 ± 7.25Ab	124.22 ± 14.02Aa	81.67 ± 5.83Ab
	10–20	47.39 ± 4.60Bb	66.32 ± 4.36Ba	47.78 ± 6.53Bb
MBC (mg/kg)	0–10	377.20 ± 25.95Aa	361.21 ± 27.88Aa	318.49 ± 23.73Ab
	10–20	336.61 ± 23.06Ba	301.84 ± 34.85Bb	271.68 ± 15.86Bb
MBN (mg/kg)	0–10	93.80 ± 7.34Aa	78.15 ± 6.40Ab	87.13 ± 6.02Ac
	10–20	73.39 ± 6.83Ba	67.42 ± 7.22Bb	64.80 ± 6.60Bc
NH_4_^+^-N (mg/kg)	0–10	71.77 ± 2.63Aa	65.02 ± 2.37Ab	60.37 ± 3.13Ac
	10–20	41.43 ± 2.25Ba	35.64 ± 3.25Bb	30.82 ± 1.87Bc
NO_3_^–^-N (mg/kg)	0–10	4.48 ± 0.12Ac	5.05 ± 0.21Ab	6.10 ± 0.34Aa
	10–20	2.87 ± 0.11Bb	3.05 ± 0.19Bb	4.23 ± 0.43Ba

Values in the table are mean ± standard deviation (SD). Different lowercase letters indicate significant differences between different fire intensities in the same soil layer (*P* < 0.05); Different uppercase letters indicate significant differences between different soil layers of the same fire intensity (*P* < 0.05).

### 3.2 Soil microbial community

#### 3.2.1 Alpha and beta diversity

The Shannon index was adopted to assess community diversity, whereas the Sobs and Chao1 indices measured community richness, and the coverage index reflected the extent of community coverage. The α-diversity indices for soil bacterial and fungal communities under different fire intensities are presented in Table 5. In the severely burned plot, the Shannon index for bacterial communities in the 10–20 cm soil layer was significantly different from that of the unburned plot (*P* < 0.05), whereas the Chao1 index in the 0–10 cm soil layer was significantly lower than that in the other treatments (*P* < 0.05). For fungal communities, the Shannon index in the 10–20 cm soil layer was higher than that in the 0–10 cm layer, with a significant difference between these layers in the severely burned plot (*P* < 0.05). Additionally, the Sobs and Chao1 indices were significantly different between the two soil layers in severely burned plots (*P* < 0.05). The 10–20 cm lightly burned plot also showed significant differences compared with the other burned plots (*P* < 0.05).

**TABLE 5:** Effect of fire intensity on the alpha diversity of soil microorganisms.

Microbe sort	Soil layer	Fire intensity	Shannon index	Sobs index	Chao1 index	Coverage index
Bacteria	0–10 cm	Unburned	5.23 ± 0.07Aa	897.83 ± 31.89Aa	1003.48 ± 30.28Aa	0.99 ± 0.00Ab
		Light fire	5.28 ± 0.09Aa	918.50 ± 41.78Aa	1013.64 ± 37.08Aa	0.99 ± 0.00Ab
		Severe fire	5.03 ± 0.10Aa	727.00 ± 62.19Ab	815.29 ± 70.74Ab	0.99 ± 0.00Aa
	10–20 cm	Unburned	5.41 ± 0.09Aa	934.17 ± 24.97Aa	1042.96 ± 20.05Aa	0.99 ± 0.00Aa
		Light fire	5.39 ± 0.04Aab	911.83 ± 14.75Aa	1018.53 ± 19.80Aa	0.99 ± 0.00Aa
		Severe fire	5.13 ± 0.11Ab	835.50 ± 57.96Aa	943.78 ± 64.19Aa	0.99 ± 0.00Aa
Fungal	0–10 cm	Unburned	2.87 ± 0.29Aa	242.17 ± 15.70Aa	295.79 ± 23.01Aa	1.00 ± 0.00Ab
		Light fire	2.96 ± 0.22Aa	237.50 ± 21.99Aa	274.06 ± 24.24Aa	1.00 ± 0.00Bab
		Severe fire	2.65 ± 0.21Ba	200.33 ± 13.54Ba	227.94 ± 17.50Ba	1.00 ± 0.00Aa
	10–20 cm	Unburned	3.35 ± 0.15Aa	247.17 ± 9.89Aa	282.95 ± 11.78Aa	1.00 ± 0.00Aa
		Light fire	3.09 ± 0.10Aa	211.00 ± 9.35Ab	240.83 ± 5.51Ab	1.00 ± 0.00Aa
		Severe fire	3.44 ± 0.10Aa	252.17 ± 10.99Aa	280.59 ± 12.00Aa	1.00 ± 0.00Aa

Values in the table are mean ± standard deviation (SD). Different lowercase letters indicate significant differences between different fire intensities in the same soil layer (*P* < 0.05); Different uppercase letters indicate significant differences between different soil layers of the same fire intensity (*P* < 0.05).

**FIGURE 2:**
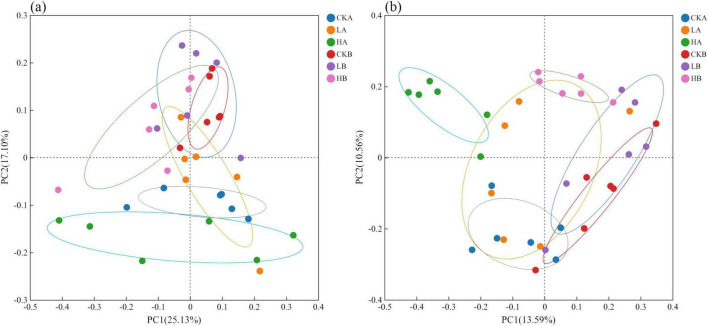
Principal Coordinate Analysis (PCoA) analysis of soil microbial communities under different fire intensities. **(a)** Pcoa of bacteria; **(b)** Pcoa of fungal. CK, not burned; L, Light fire; H, severe fire; A, 0–10 cm; B, 10–20 cm. The following figures use the same notes.

Principal Coordinate Analysis (PCoA) based on Bray-Curtis similarity was used to assess bacterial and fungal community structures. The PCoA results for the bacterial community (Figure 2a) indicated that PC1 and PC2 contributed 25.13% and 17.10% of the variation, respectively. Significant differences were observed between the 0 and 10 cm soil layers of the severely burned and unburned plots (*P* < 0.05), as well as between different soil layers within the severely burned plots (*P* < 0.05). However, the differences in the community structure between different fire intensities were minimal, suggesting that a light fire had no significant impact on the bacterial community structure. The PCoA results for the fungal community (Figure 2b) showed that PC1 and PC2 contributed to 13.59% and 10.56% of the variation, respectively. Under the same fire intensity, the differences among soil layers were minor, and the fungal community variations closely mirrored those of the bacterial communities. Additionally, the fungal communities across different fire intensity plots showed no significant differences.

#### 3.2.2 Soil microbial community structure

**FIGURE 3:**
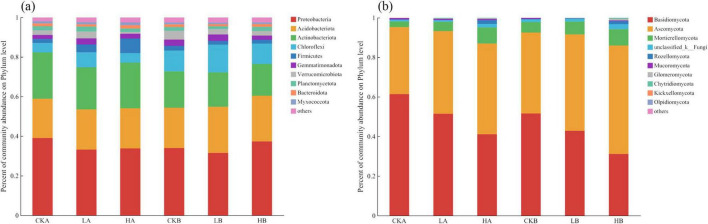
Community composition of soil bacteria and fungi at the phylum level under different fire intensities. **(a)** Phylum level community composition of bacteria; **(b)** Phylum level community composition of fungal.

Sequencing of soil bacteria across three different fire intensity plots in the *L. gmelinii* forests identified 26 phyla, 57 classes, 125 orders, 188 families, and 294 genera. The top 10 bacterial phyla accounted for over 98% of the bacterial communities in the soil samples, with Proteobacteria, Acidobacteria, Actinobacteria, Chloroflexi, and Firmicutes collectively representing more than 90% of the total abundance. Proteobacteria was the most abundant phylum across unburned, lightly burned, and severely burned plots in different soil layers. In the 0–10 cm soil layer, bacterial abundance accounted for approximately 39.10%, 33.22%, and 33.83% in the unburned, lightly burned, and severely burned plots, respectively, whereas in the 10–20 cm layer, these values were 34.03%, 31.54%, and 37.37%, respectively (Figure 3a). The relative abundance of Acidobacteria was not significantly different across fire intensities. However, it was higher in the 0–10 cm layer of the burned plots than in the unburned plots, whereas in the 10–20 cm layer, its abundance was lower than that in the unburned plots. With increasing soil depth, the relative abundance of Acidobacteria and Actinobacteria gradually increased, whereas Chloroflexi showed a decreasing trend.

Sequencing of soil fungal across the three fire intensity plots in the *L. gmelinii* forests identified 11 phyla, 34 classes, 71 orders, 128 families, and 194 genera. Among the fungal communities, three phyla, including Basidiomycota, Ascomycota, and Mortierellomycota, had an average relative abundance exceeding 2%, with Basidiomycota and Ascomycota together accounting for over 90% of the total fungal OTUs (Figure 3b). The relative abundance of the three in the 0–10 cm soil layer was ranked from small to large: unburned (41.14%), light fire (51.48%), and severe fire (61.39%). In the 10–20 cm layer, the ranking was severe (31.16%), light (42.89%), and unburned (51.62%). With increasing fire intensity, the relative abundance of Ascomycota gradually increased, whereas that of Basidiomycota showed a decreasing trend.

**FIGURE 4:**
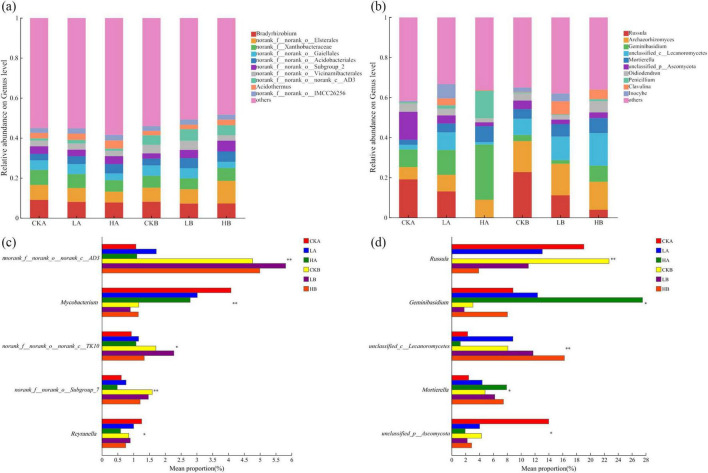
The community composition of soil bacteria and fungi at the genus level under different fire intensities. **(a)** Genus level community composition of bacteria; **(b)** Genus level community composition of fungal; **(c)** Comparative analyses of differences in genus level community composition of bacteria; **(d)** Comparative analyses of differences in genus level community composition of fungal. * and ** indicate *P* < 0.05 and *P* < 0.01, respectively.

At the level of bacterial genus, it was found that the relative abundance of all bacterial genera was less than 12%, the number of bacterial genera was large, and the proportion of unnamed bacteria was high (Figure 4a). The sequences that cannot be classified to the known genus level are uniformly classified into “others”. The top five dominant genera at the bacterial genus level in the three burned plots were *Bradyrhizobium*, *norank_f__norank_o__Elsterales*, *norank_f__Xanthobacteraceae*, *norank_f__norank_o__Gaiellales* and *norank_f__norank_o__Acidobacteriales*, and the sum of relative abundance accounted for 11.85%–25.50% of the total abundance of soil bacteria. The relative abundance of Bradyrhizobium in the soil surface layer from large to small was unburned (9.09%), light fire (8.07%), and severe fire (7.79%). The underlying soil was unburned (8.13%), severely burned (7.23%), and lightly burned (7.26%). The relative abundance of *norank_f__norank_o__Gaiellales* (5.00%) and *norank_f__norank_o__Acidobacteriale* (3.94%) after light burning was higher than that before burning. After severe fire, only the relative abundance of *norank_f__norank_o__Acidobacteriales* (4.71%) was higher than that of unburned plots. The results showed that *nnorank_f__norank_o__norank_c__AD3*, *Mycobacterium* and *norank_f__norank_o__Subgroup_7 had* extremely significant effects on the two soil layers of three fire intensities (*P* < 0.01) (Figure 4c).

At the fungal genus level, the sequences that could not be classified at the known genus level were uniformly classified as “others,” and most of them were classified as *Russula* (0.07%–22.69%), *Archaeorhizomyces* (6.12%–15.80%), *Geminibasidium* (1.81%–27.52%). Followed by *unclassified_c__Lecanoromycetes* (2.46%–7.93%) and *Mortierella* (1.94%–13.99%) (Figure 4b). With the increase of fire intensity, the relative abundance of *Russula* decreased gradually, and *Archaeorhizomyces* and *Geminibasidium* were dominant under severe fire intensity. After different fires, *Archaeorhizomyces* and *unclassified_c__Lecanoromycetes* increased with the increase of soil depth, and *unclassified_c__Lecanoromycetes* had extremely significant differences in the two soil layers (*P* < 0.01) (Figure 4d). *Russula* and *Geminibasidium* decreased with the increase of soil depth, and there were significant or extremely significant differences between the two soil layers (*P* < 0.05, *P* < 0.01). In general, the relative abundance of dominant genera of soil fungal community fluctuated greatly after different fire intensities, and the uncertainty of its change was higher, which was more sensitive than that of bacterial community.

### 3.3 Functional genes in the soil microbial nitrogen cycle

**FIGURE 5:**
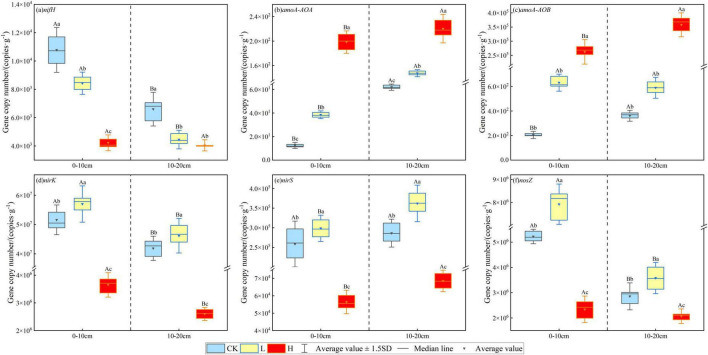
Effects of different fire intensities on the abundance of soil nitrogen cycle functional genes.

As shown in Figure 5, the abundance of soil microbial nitrogen cycle functional genes varied significantly across the fire intensity plots. The abundance of the *nifH* gene decreased with increasing fire intensity, with reductions of 21.83% and 32.59% in the 0–10 cm and 10–20 cm soil layers of the light fire plots, respectively, and 60.84% and 38.73% in the severe fire plots, respectively, compared with the unburned plots (*P* < 0.05). In contrast, the abundance of *amoA-AOA* and *amoA-AOB* genes increased with fire intensity, with severe fires causing a significantly greater increase than light fires, demonstrating significant differences across soil layers (*P* < 0.05). Light fires increased the abundance of denitrifying *nirK*, *nirS*, and *nosZ* genes, whereas severe fires caused a decline. Among them, the *nirK* gene was the most affected, decreasing by 92.92% and 93.79% in the 0–10 cm and 10–20 cm soil layers, respectively, compared with the unburned plots (*P* < 0.05).

### 3.4 Effect mechanism of fire intensity on soil microbial community and nitrogen cycle

The nitrogen fixation *nifH* gene and denitrification *nirK* and *nirS* genes exhibited significant negative correlations with BD (*P* < 0.01), whereas the *nosZ* gene showed a significant negative correlation with BD (*P* < 0.05) (Table 6). These four genes were significantly positively correlated with TN (*P* < 0.01). In contrast, the nitrifying *amoA-AOA* and *amoA-AOB* genes were significantly positively correlated with BD (*P* < 0.01) and significantly negatively correlated with TN (*P* < 0.01). Nitrogen fixation, nitrification, and denitrification genes were significantly correlated with MBC (*P* < 0.05), whereas all genes, except *nirS*, showed significant correlations with SOC (*P* < 0.05). The *nifH* gene showed a significant positive correlation with MBN (*P* < 0.01), with a correlation coefficient of 0.548. The *amoA-AOA* gene was significantly negatively correlated with MBN and NH_4_^+^-N (*P* < 0.01), with correlation coefficients of −0.418 and −0.597, respectively. The *amoA-AOB* gene exhibited significant correlations with NH_4_^+^-N and NO_3_^–^-N (*P* < 0.05), with correlation coefficients of −0.328 and 0.439, respectively.

**TABLE 6:** Correlation coefficients between soil physicochemical properties and nitrogen cycling gene abundance.

Gene	pH	BD	SMC	TN	AP	AK	SOC	MBC	MBN	NH_4_^+^−N	NO_3_^–^−N
*nifH*	−0.310*	−0.408^**^	0.092	0.439^**^	−0.098	−0.184	0.526^**^	0.783^**^	0.548^**^	0.724	0.035
*nirS*	−0.198	−0.650^**^	0.306*	0.754^**^	−0.469^**^	−0.661^**^	0.227	0.351*	−0.087	0.055	−0.610*
*nirK*	−0.211	−0.635^**^	0.179	0.808^**^	−0.300*	0.524^**^	0.473^**^	0.584^**^	0.145	0.360*	−0.387*
*nosZ*	−0.025	−0.378*	−0.205	0.794^**^	0.148	−0.147	0.851^**^	0.612^**^	0.260	0.625^**^	0.173
*amoA-AOA*	0.259	0.552^**^	−0.157	−0.578^**^	0.296*	0.339*	−0.483^**^	−0.765^**^	−0.418^**^	−0.597^**^	0.188
*amoA-AOB*	0.151	0.589^**^	−0.249	−0.709^**^	0.367*	0.547*	−0.350*	−0.585*	−0.185	−0.328*	0.439^**^

* represents *P* < 0.05, and ** represents *P* < 0.01.

**FIGURE 6:**
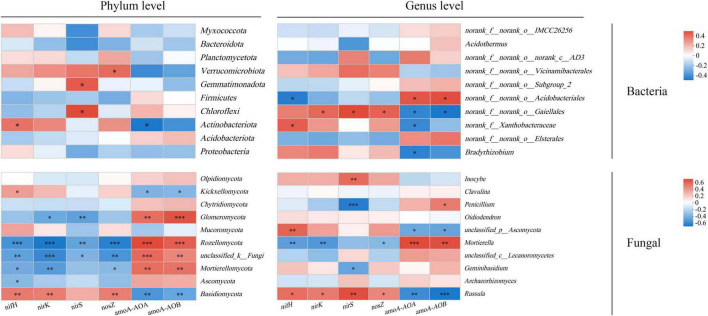
Correlation analysis between dominant bacteria at phylum and genus levels of soil microbial community and functional genes of soil microbial nitrogen cycle. *, ^**^, and ^***^ indicate *P* < 0.05, *P* < 0.01, and *P* < 0.001, respectively.

In the bacterial community, the *nifH* gene showed a significant positive correlation with Acidobacteria, the *nirS* gene was positively correlated with Chloroflexi and Gemmatimonadetes, and the *nosZ* gene exhibited a significant positive correlation with Verrucomicrobia (*P* < 0.05) (Figure 6). Conversely, the *amoA-AOB* gene showed a significant negative correlation with Acidobacteria (*P* < 0.05). In the fungal community, *nifH*, *nirK*, and *nosZ* were significantly positively correlated with Mortierella, Rhodomycota, and unclassified_k_Fungi (*P* < 0.01). The *nirS* gene was significantly correlated with Rhodomycota, unclassified_k_Fungi, and Glomeromycota (*P* < 0.01). The *amoA-AOA* and *amoA-AOB* genes showed significant negative correlations with Mortierella, Rhodobacter, unclassified_k_Fungi, and Glomeromycota (*P* < 0.01). Except for *nirS*, all other genes were significantly correlated with Basidiomycota (*P* < 0.05). Ascomycota was only significantly associated with the *nifH* gene (*P* < 0.05), whereas Chytridiomycota showed no significant correlation with any gene.

At the genus level, the *nifH* gene in the bacterial microbial community was significantly correlated with *norank_f__Xanthobacteraceae* and *norank_f__norank_o__Acidobacteriales* (*P* < 0.05). Except for *nifH* gene, *norank_f__norank_o__Gaiellales* was significantly correlated with other genes (*P* < 0.05). The *amoA-AOA* gene and *amoA-AOB* gene were significantly correlated with *norank_f__norank_o__Gaiellales* and *norank_f__norank_o__Acidobacteriales* (*P* < 0.05). In addition, *amoA-AOA* gene was significantly negatively correlated with *Bradyrhizobium* and *norank_f__Xanthobacteraceae* (*P* < 0.05). In the fungal microbial community, *Russula* was significantly negatively correlated with *amoA-AOA* and *amoA-AOB* genes (*P* < 0.01), and positively correlated with other genes (*P* < 0.05). Except for nirS gene, the other genes were significantly correlated with *Mortierella*, and were significantly positively correlated with nitrification genes (*P* < 0.01). The *nirS* gene was significantly correlated with *Russula*, *Penicillium* and *Inocybe* (*P* < 0.01).

**FIGURE 7:**
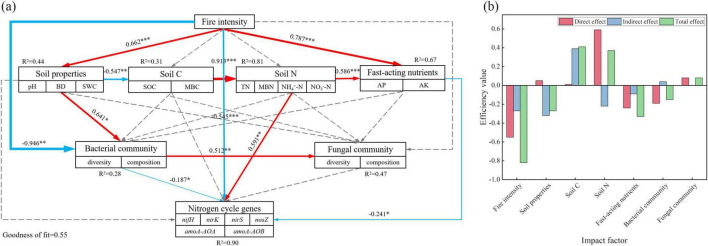
Path analysis based on the partial least squares path model (PLS-PM). **(a)** Path analysis model; **(b)** Efficiency value. illustrating the effects of soil physicochemical properties and microbial diversity on the abundance of nitrogen cycle functional genes, as well as the direct and indirect effects of each influencing factor. The thickness of the arrow represents the level of correlation. The red solid arrow indicates a significant positive correlation, the blue solid arrow indicates a significant negative correlation, the dotted arrow indicates no correlation, and *R*^2^ indicates path interpretation. *, **, and *** indicate *P* < 0.05, *P* < 0.01, and *P* < 0.001, respectively.

The correlation heatmap and table of correlation coefficients illustrate the relationships among soil physicochemical properties, microbial community structure, and nitrogen cycle gene abundance. To further analyze the impact of forest fire disturbance on nitrogen cycle functional genes, a PLS-PM path model was applied (Figure 7a). The results indicated that fire and available nutrients had direct negative effects on the nitrogen cycle, with path coefficients of −0.545 and −0.241, respectively. In contrast, soil nitrogen had a direct positive effect on the nitrogen cycle, with a path coefficient of 0.591. Fire also significantly influenced the available nutrients (*P* < 0.001), with a path coefficient of 0.787, indirectly reducing nitrogen cycle gene abundance. Additionally, fire directly affected the soil properties (*P* < 0.001) with a path coefficient of 0.662, which positively influenced bacterial community, and the path coefficient was 0.641 (*P* < 0.05), ultimately affecting the nitrogen cycle genes (path coefficient = −0.187, *P* < 0.05). Moreover, the fire indirectly affected soil carbon and nitrogen through its influence on soil properties, leading to changes in available nutrients and a subsequent negative impact on nitrogen cycle genes. As shown in Figure 7b, fire had the strongest overall effect on the microbial nitrogen cycle genes, whereas the soil carbon and nitrogen, and available nutrients are the key factors influencing the nitrogen cycle gene abundance.

## 4 Discussion

### 4.1 Effects of burning intensity on soil physical and chemical properties

Forest fires can alter forest soil properties by consuming litter layers through oxidation, volatilization, ash convection, and leaching (Pellegrini et al., 2018; Lou et al., 2023). This study observed that soil pH increased with fire intensity, which was consistent with the pH value conclusion of Akburak et al. (2018). This occurred because at soil temperatures above 200°C, combustion could form coke-like compounds and ash, which are rich in alkaline cations that raise the soil pH (Agbeshie et al., 2022). The increase in soil BD after a fire was due to the destruction of soil organic matter and aggregates, as well as the mixing of ash and partially combusted plant residues, which may clog soil pores (Zhao et al., 2023; Verma et al., 2019). SMC was lower in the burned plots, likely due to reduced vegetation cover, increased direct sunlight exposure, accelerated soil moisture evaporation, and pore blockage, which lowered the water retention capacity (Xue et al., 2011; Zavala et al., 2014). This study suggested that the SOC content increased in the lightly burned plots, consistent with Liu et al. (2018), as the light fire enhanced the soil temperature, stimulated microbial activity, and promoted the decomposition of unburned organic matter, increasing the SOC levels (Zhang et al., 2018). However, SOC decreased in the severely burned plots, likely due to the combustion of Soil Organic Matter (SOM), carbon mineralization, volatilization, increased carbon dissolution from nutrient-rich ash, and high pH (Rodríguez-Cardona et al., 2020). Fire significantly affected the TN content, which increased in the lightly burned plots (Table 4) because the fire converted soil organic nitrogen into inorganic nitrogen, and the resulting NH_4_^+^-N could bind to the negatively charged minerals and organic matter, leading to nitrogen retention (Scharenbroch et al., 2012). The NO_3_^–^-N content increased with the fire intensity, aligning with Goberna et al. (2012), while NH_4_^+^-N decreased due to the high temperatures converting NH_4_^+^-N into ammonia (NH_3_^–^-N), which can volatilize into the atmosphere (Zhang et al., 2017), consistent with Shi et al. (2022). Additionally, fire significantly increased AP and AK, likely due to nutrient leaching from vegetation and organic ash into the soil, enriching the nutrient content (Rivelli and Libutti, 2022).

### 4.2 Effects of fire intensity on soil microbial community diversity and composition

The analysis of Shannon, Sobs, Chao1, and Coverage indices revealed that different fire intensities had varying effects on the soil microbial community diversity (Table 5). High-intensity and long-duration fires have the most pronounced impact on surface soil microorganisms, whereas their influence on deeper soil microbial communities is minimal (Bastias et al., 2006). This study demonstrated that a light fire enhanced the microbial diversity in the surface soil (0–10 cm), whereas a severe fire significantly reduced the diversity and richness compared with the unburned plots. In particular, the bacterial diversity in severely burned plots declined sharply, likely due to high temperatures damaging the bacterial cell structures and functions, leading to a substantial loss of microbial diversity (Cheng et al., 2024). Additionally, severe fire increased the surface soil pH, altered microbial habitat, and created unfavorable conditions for microbial activity, further reducing diversity and richness while maintaining consistent community coverage. These findings indicated that severe fire significantly decreased microbial diversity and richness but did not affect community coverage.

Burning significantly altered the β-diversity of soil microorganisms, with the bacterial community composition differing significantly across severely burned sites, consistent with previous studies (Shu et al., 2022). The study revealed that the microbial community structure in the 0–10 cm soil layer of the lightly burned plot closely resembled that of the unburned plot, whereas that of the heavily burned plot was significantly different from that of the unburned plot (Figure 2). This suggested that severe fires had a more pronounced impact on surface soil microorganisms. The likely cause was that fire could burn the forest canopy to varying degrees, leading to increased Soil Temperature(ST), reduced SMC, and shifts in nutrient availability, which in turn affected BD, soil porosity, and ultimately the microbial community structure (Yang et al., 2017; Liu et al., 2021).

Fires can change the physical and chemical properties of soil and the forest microenvironment (Hong et al., 2010), indirectly affecting the survival of soil microorganisms and reshaping microbial community composition (Huang et al., 2022). In this study, Proteobacteria, Acidobacteria, Actinobacteria, and Chloroflexi were the dominant bacterial phyla, consistent with previous research (Li et al., 2024). Among these, Proteobacteria was the most abundant, containing plant probiotics that play a key role in the soil nitrogen cycle (Wang et al., 2020). Notably, this study found that the relative abundance of soil bacteria decreased following a light fire, in contrast to the findings of Ward et al. (2009). This decline may be due to the reduction in soil organic matter content after the fire, which probably lowered hydrolase activity and subsequently affected bacterial activity and abundance (Pei et al., 2023). However, in the severely burned plot, bacterial abundance increased in the 10–20 cm soil layer, suggesting that fire disturbance can promote bacterial community diversity at greater depths (She et al., 2021). Compared to the unburned plots, the Acidobacteria abundance increased in burned plots, likely because the fire-induced changes in soil physicochemical properties and the forest microenvironment indirectly influenced microbial survival and community composition (Dove et al., 2022). At the genus level, most of the bacteria with relative abundance > 1% were uncultured bacteria, and only a few were identified. Since the abundance of significant bacterial genera in the community was less than 5%, the subsequent analysis only focused on the dominant genera with higher abundance (Figure 4c). The relative abundance of bacteria in each soil layer was evenly distributed under different fire intensities, indicating that the fire had little effect on the composition of fungi in the two soil layers (Li et al., 2019).

The results showed that Basidiomycota and Ascomycota were the dominant fungal phyla regardless of fire intensity and soil layer. Although different fire intensities affected their relative abundance, they did not change the pattern of dominant phyla. The combined relative abundance of Ascomycota and Basidiomycota exceeded 90% across all plots, which was consistent with previous findings (Ammitzboll et al., 2021). In this study, Basidiomycota had the highest relative abundance in the unburned plots and progressively declined with increasing fire intensity, aligning with the results of Bergner et al. (2004). Conversely, Ascomycota abundance increased with fire intensity, which may be attributed to the role of Basidiomycota in lignin decomposition (Lienhard et al., 2014). However, as Basidiomycota coexisted with plant roots, its resistance to environmental disturbances was lower than that of Ascomycota, which may explain the observed shift in fungal community composition (Wang et al., 2022). At the level of fungal genus classification, *Russula* is the dominant group, which expands the absorption range of plant roots by forming a symbiotic relationship with plant roots, thus promoting the uptake of nitrogen by plants. In addition, it can also absorb inorganic nitrogen from the soil and convert it into organic nitrogen forms available to plants, regulate the activity of nitrogen cycle-related enzymes, and affect the soil nitrogen transformation process (Hestrin et al., 2019). *Geminibasidium* can decompose lignin and cellulose to release nitrogen, increase nitrogen content in soil, increase soil fertility, and provide available nitrogen sources for plants and other microorganisms (Ren et al., 2023). In addition, the relative amplitude of soil fungal community in *Larix gmelinii* forest after fire fluctuated widely (Figure 4b), indicating that fungi were more sensitive to forest fire than bacteria (Holden et al., 2013).

### 4.3 Effects of fire intensity and related factors on the abundance of soil microbial nitrogen cycling functional genes

This study indicated that the soil microbial nitrogen cycle was primarily driven by organic nitrogen synthesis, nitrification, and denitrification, and that fire intensity significantly affected the abundance of nitrogen cycle-related genes in the microbial community. The abundance of the nitrogen-fixing *nifH* gene decreased as the fire intensity increased, indicating that a higher fire intensity negatively affected the nitrogen-fixing microbial populations, potentially reducing the soil nitrogen fixation capacity. Additionally, the composition of nitrogen-fixing microorganisms is influenced by microenvironmental factors such as vegetation type, soil physicochemical properties, nutrient availability, and hydrothermal conditions (Poly et al., 2001). Correlation analysis revealed that the *nifH* gene was significantly positively correlated with soil pH, BD, TN, SOC, MBC, and MBN (Table 6), suggesting that the fire indirectly affected *nifH* gene abundance by altering soil physicochemical properties. Spearman’s correlation analysis further showed a significant positive correlation between *nifH* and Basidiomycota genes (Figure 5). Because Basidiomycota species form mycorrhizal symbioses with plants, facilitating nutrient uptake and growth, they may be linked to *nifH* gene expression, potentially playing a role in plant-associated nitrogen fixation (Yang Y. et al., 2021).

The abundance of nitrifying *amoA-AOA* and *amoA-AOB* genes increased with fire intensity, likely due to post-fire changes in soil conditions, such as temperature, nutrient availability, and permeability, which create a more favorable environment for nitrifying bacteria (DeLuca et al., 2006; Xu et al., 2018). These genes were significantly positively correlated with AP and AK, consistent with the findings of Zhang et al. (2022). Phosphorus can be essential for bacterial ATP synthesis and other critical phosphorus-containing compounds (Fang et al., 2023), while potassium can regulate the enzyme activity and intracellular signaling (Wang et al., 2021), contributing to the increased abundance of *amoA-AOA* and *amoA-AOB* genes. Additionally, these genes were significantly negatively correlated with Mortierella, Rhodomycota, and Glomeromycota. This may be attributed to the substantial depletion of organic matter and nutrients in the post-fire soil, intensifying resource limitations. In such environments, competition between bacteria and fungi may arise with ammonia-oxidizing bacteria harboring *amoA-AOA* and *amoA-AOB* genes gaining a competitive advantage, thereby suppressing the abundance of fungal phyla (Yang et al., 2024).

Denitrification is a biological process in which soil NO_3_^–^ is reduced to NO_2_^–^, NO, N_2_O, and N_2_ under anaerobic conditions and serves as a primary pathway for soil nitrogen loss (Coyne et al., 2018). This study suggested that the denitrifying *nirK*, *nirS*, and *nosZ* genes were significantly reduced after a severe fire, likely because the high post-fire temperatures impaired the metabolic activity of denitrifying bacteria, thereby reducing their ability to decompose nitrate and lowering the expression of denitrification-related genes (Feng et al., 2023). TN was significantly positively correlated with these three genes, as increased TN levels could provide more energy and nitrogen sources for denitrifying bacteria, promoting the expression and abundance of *nirK*, *nirS*, and *nosZ* genes (Yang Q. et al., 2020). Additionally, *nirS* was positively correlated with Chloroflexi and Gemmatimonadetes, suggesting that changes in the soil nitrogen pool (TN and NH_4_^+^-N), C availability (MBC and readily oxidizable carbon), and microclimate (SMC and ST) significantly influenced the structure and diversity of *nirS*-denitrifying bacterial communities (Cao et al., 2021). These environmental changes may enhance *nirS* gene expression while also supporting the growth of Chloroflexi and Gemmatimonadetes. The model has been widely used in forest fires (Liu et al., 2024). The PLS-PM path model analysis further revealed that fire was a dominant factor affecting soil microbial nitrogen cycling, influencing nitrogen cycle gene expression by altering the soil carbon and nitrogen levels and fungal diversity, thereby regulating the nitrogen cycling processes and accelerating soil nitrogen and energy transformation.

## 5 Conclusion

The findings revealed that a light fire significantly affected the TN, SOC, and AP levels, increased the bacterial community diversity, and enhanced the fungal diversity in the surface layer, while reducing it in the bottom layer. In contrast, severe fire had a pronounced impact on AP, AK, NH_4_^+^-N, and NO_3_^–^-N, leading to a decline in bacterial diversity, while fungal diversity exhibited an opposite trend to that observed under light fire. Both fire intensities reduced microbial richness. The dominant bacteria of soil bacteria after fire were Proteobacteria, Acidobacteria and Actinobacteria, and *Bradyrhizobium* was the dominant genus. The dominant phyla of fungi were Basidiomycetes, Ascomycota and Mortierella, and *Russula* was the dominant genus. Fire directly influenced the nitrogen cycling processes, particularly affecting the abundance of nitrifying *amoA-AOA* and *amoA-AOB* genes, or indirectly altering the soil properties (pH, BD, and SMC), soil carbon and nitrogen (SOC, MBC, MBN, TN, NH_4_^+^-N, and NO_3_^–^-N), available nutrients (AP and AK), and microbial diversity. Basidiomycetes, Mortierella, Rhodomycota, and Glomeromycota were the key fungal phyla involved in the nitrogen cycle, *Russula* and *Mortierella* are the main fungal genera that affect the soil microbial nitrogen cycle. These findings provide a scientific foundation for the effective management of vegetation recovery, regeneration, and nitrogen pool reconstruction following wildfires.

## Data Availability

The original contributions presented in this study are publicly available. The raw microbiome data can be found here: https://www.ncbi.nlm.nih.gov/sra, accession numbers SRP499782, SRP499761, and SRP581037.

## Footnotes

1
https://github.com/OpenGene/fastp

2
http://www.cbcb.umd.edu/software/flash

3
http://drive5.com/uparse/

4
https://cloud.majorbio.com
